# Comparison between superior vena cava ablation in addition to pulmonary vein isolation and standard pulmonary vein isolation in patients with paroxysmal atrial fibrillation with the cryoballoon technique

**DOI:** 10.1007/s10840-020-00932-6

**Published:** 2021-01-15

**Authors:** Ingrid Overeinder, Thiago Guimarães Osório, Paul-Adrian Călburean, Antonio Bisignani, Gezim Bala, Juan Sieira, Erwin Ströker, Maysam Al Houssari, Joerelle Mojica, Serge Boveda, Gaetano Paparella, Pedro Brugada, Carlo de Asmundis, Gian-Battista Chierchia

**Affiliations:** Heart Rhythm Management Center, Postgraduate program in Cardiac Electrophysiology and Pacing, European Reference Networks Guard-Heart, Universitair Ziekenhuis Brussel-Vrije Universiteit Brussel, Laarbeeklaan 101, Brussels, Belgium

**Keywords:** Superior vena cava isolation, Paroxysmal atrial fibrillation, Pulmonary vein isolation, Second-generation cryoballoon

## Abstract

**Background:**

Paroxysmal atrial fibrillation (PAF) can be triggered by non-pulmonary vein foci, like the superior vena cava (SVC). The latter is correlated with improved result in terms of freedom from atrial tachycardias (ATs), when electrical isolation of this vessel utilizing radiofrequency energy (RF) is achieved.

**Objectives:**

Evaluate the clinical impact, in patients with PAF, of the SVC isolation (SVCi) in addition to ordinary pulmonary vein isolation (PVI) by means of the second-generation cryoballoon (CB)

**Methods:**

A total of 100 consecutive patients that underwent CB ablation for PAF were retrospectively selected. Fifty consecutive patients received PVI followed by SVCi by CB application, and the following 50 consecutive patients received standard PVI. All patients were followed 12 months.

**Results:**

The mean time to SVCi was 36.7 ± 29.0 s and temperature at SVC isolation was − 35 (− 18 to − 40) °C. Real-time recording (RTR) during SVCi was observed in 42 (84.0%) patients. At the end of 12 months of follow-up, freedom from ATs was achieved in 36 (72%) patients in the PVI only group and in 45 (90%) patients of the SVC and PV isolation group (Fisher’s exact test *p* = 0.039, binary logistic regression: *p* = 0.027, OR = 0.28, 95%CI = 0.09–0.86). In survival analysis, SVC and PV isolation group was also associated with improved freedom from ATs (log-rank test: *p* = 0.017, Cox regression: *p* = 0.026, HR = 0.31, 95%CI = 0.11–0.87).

**Conclusion:**

Superior vena cava isolation with the CB in addition to PVI might improve freedom from ATs if compared to PVI alone at 1-year follow-up.

## Introduction

Paroxysmal atrial fibrillation (PAF) can be triggered by non-pulmonary vein foci, like the superior vena cava (SVC). The latter is correlated with improved result in terms of freedom from atrial tachycardias (ATs) when electrical isolation of this vessel utilizing radiofrequency energy (RF) is achieved [1–3]. As published more than a decade ago, patients with PAF were less prone to experience recurrence with RF, after a follow-up of 12 months when the isolation of the SVC was add in patients with PAF who underwent pulmonary vein isolation (PVI), compared with the group that received PVI solely [1]. In this study, we describe a retrospective study in a cohort of consecutive patients undergoing PVI or PVI + SVC ablation with the second-generation cryoballoon (CB).

## Methods

### Study design

Consecutive patients programmed for CB ablation for PAF were retrospectively enrolled in our study. After PVI was obtained, if the SVC exhibited electrical activity, isolation was accomplished through a single 180-s duration cryoenergy application, which is recognized to create a long-lasting lesion [4]. Phrenic nerve injury (PNI), although nearly always reversible, is the most frequently observed complication during CB ablation [5]. To avoid the latter, a decapolar catheter was inserted through the right jugular/subclavian to allow simultaneous ablation in the SVC and phrenic nerve (PN) pacing.

### Patient selection

Consecutive patients programmed for CB ablation for PAF were enrolled between August 2018 and November 2018. All antiarrhythmic drugs were discontinued at least 3 days before ablation, apart from amiodarone which was stopped 1 month before. Procedures were done under general anesthesia. The study was approved by the ethical committee. The protocol was carried out in accordance with the ethical principles for medical research involving human subjects established by the Declaration of Helsinki, protecting the privacy of all participants as well as the confidentiality of their personal information. The exclusion criteria were any contraindications for the procedure, including the presence of an intracavitary thrombus, uncontrolled heart failure, contraindications to general anesthesia, and prior AF ablation.

### Procedure

#### Pulmonary vein isolation

As previously described [6], after having obtained LA access, through a steerable 15 Fr sheath (FlexCath Advance Medtronic Inc., Minneapolis, MN, USA), a 28-mm CB-A (Arctic Front Advance, Medtronic Inc., Minneapolis, MN, USA) was advanced in the LA and an inner lumen mapping catheter (MC; Achieve, Medtronic Inc., Minneapolis, MN, USA) was positioned in each PV ostium. Baseline electrical information was gathered in each PV ostium. The 28-mm CB-A was advanced, inflated, and positioned at each PV ostium. Optimal vessel occlusion was defined by selective contrast injection showing total contrast retention with no backflow into the LA. The ablation sequence was treating the left superior PV (LSPV) first, followed by the left inferior PV (LIPV), right inferior PV (RIPV), and right superior PV (RSPV). Once vessel occlusion was deemed satisfactory, delivery of cryoenergy to allow freezing was commenced. Standard cryothermal applications lasted 180 s. Our target temperature was − 40 °C within the first 60 s. If the temperature did not attain this value, an extra freeze was delivered. Successful PVI was defined as an absence of all PV potentials or their dissociation from an atrial activity.

#### SVC isolation

**Fig. 1:**
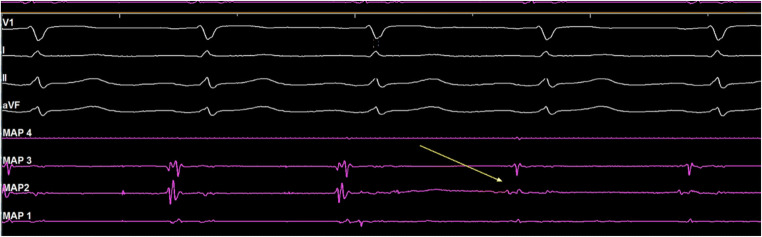
Real-time SVC electrical activity. Example of potentials recorded at the ostium of the SVC during sinus rhythm prior to isolation and electrical isolation of the SVC as measured by the circular mapping catheter (yellow arrow). Shown are surface leads V1, I, II, and AVF and bipolar intracardiac electrograms recorded by circular mapping catheter (MAP 1- 4)

**Fig. 2:**
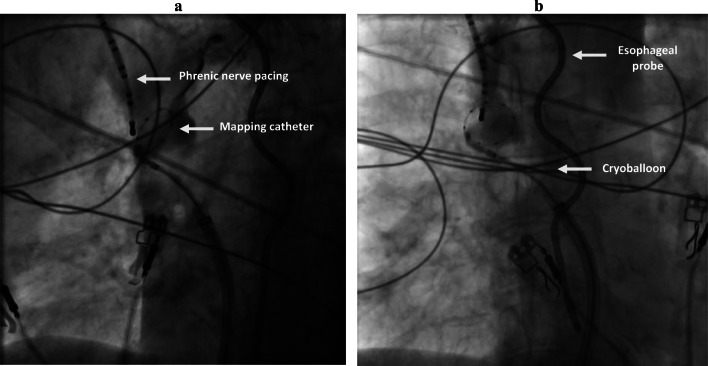
SVC isolation as seen from LAO incidence (A) and from PA incidence (B).

After PVI, the CB was retrieved to the right atrium and the achieve catheter was advanced in the SVC. Real-time SVC potentials were sought prior to cryoablation with the mapping catheter (Fig. 1). SVC venogram was performed to identify the SVC-right atrium (RA) junction. Then, in order to occlude the vessel, the CB was inflated in the right atrium and advanced towards the ostium of the SVC. After total occlusion was confirmed by dye injection with total retention of contrast in the SVC, cryoenergy application was started (Fig. 2). A temperature limit of − 60 °C was used for SVC ablation.

After the SVC isolation (SVCi), a waiting period of 15 min was taken into account, and thereafter routine pharmacological testing with adenosine and isoproterenol was performed [7] to reveal dormant conduction. Following SVC isolation, SVC-RA entry block was confirmed.

#### Phrenic nerve monitoring

Prior to ablation of the right-sided PVs and the SVC, a 6F decapolar catheter was placed distally in the SVC, and diaphragmatic stimulation was achieved by pacing the ipsilateral phrenic nerve with a 1000-ms cycle and a 20-mA output. Phrenic nerve pacing started once the temperature reached − 20 °C to avoid balloon dislodgement due to diaphragmatic contraction in the first phase of cryoenergy application. Pacing was continued throughout the entire duration of cryoenergy delivery. In cases of phrenic nerve palsy, the freeze was immediately aborted with a “double stop” technique [8] and observed for recovery.

#### Post-procedural management

Post-procedural management was performed as standard clinical practice. The next day, patients underwent a trans thoracic echocardiogram (TTE) and a chest x-ray. During the chest x-ray, a “sniff test” was performed to assess PN function. The patients were monitored under telemetry for 18 h after ablation.

A blanking period of 3 months was considered. Clinical follow-up including regular cardiological consultations and 24-h Holter ECG monitoring was performed as standard clinical practice at every 3 months for a total of 12 months of follow-up. Moreover, additional Holter monitoring was performed if any symptoms typical of recurrence following ablation appeared.

### Statistical analysis

All statistical analyses were performed using SPSS version 24.0 (SPSS Inc., Chicago, IL, USA). Categorical variables were reported as absolute and relative frequencies. Contingency tables were analyzed using Fisher’s exact test. Continuous variables were evaluated for parametric distribution using Kolmogorov-Smirnov test. Continuous variables with parametric distributions were reported as mean ± standard deviation and compared using non-paired Student’s *t* test. Continuous variables with non-parametric distributions and discrete variables were reported as median (interquartile range) and compared using Mann-Whitney test. A significance cut-off for *p* value of less than 0.05 (two-sided) and a 95% confidence interval (CI) was used. Binary logistic regression was used to assess the impact of SVCi on the presence of ATs recurrence at the end of the follow-up. Survival analysis with log-rank test and Cox regression was used to identify significant predictors of arrhythmia recurrence.

## Results

A total of 100 consecutive patients with PAF were included in the study. Of those, the first 50 consecutive patients underwent PVI followed by SVCi and the following 50 consecutive patients underwent standard PVI only. All patients were followed 12 months. Incidence of comorbidities, echocardiographic parameters, and chronic medication among groups are reported in Table 1.

**Table 1:** Baseline characteristics among study arms

	PVI and SVC isolation (*n* = 50)	PVI alone (*n* = 50)	*p* value
Males	33 (66.0%)	35 (70.0%)	0.83
Mean age	54.9 ± 11.5	55.7 ± 12.0	0.73
Mean BMI	28.6 ± 5.8	29.9 ± 4.5	0.20
Median CHA_2_-DS_2_-VASc score	1 (0–2)	1 (0–2)	0.49
CHA_2_-DS_2_-VASc score ≥ 2	15 (30.0%)	23 (46.0%)	0.15
Arterial hypertension	17 (34.0%)	24 (48.0%)	0.22
Diabetes mellitus	5 (10.0%)	6 (12.0%)	0.99
Dyslipidemia	13 (26.0%)	18 (36.0%)	0.38
Coronary artery disease	5 (10.0%)	4 (8.0%)	0.99
Valvular heart disease**	10 (20.0%)	10 (20.0%)	0.99
TIA	3 (6.0%)	4 (8.0%)	0.99
Normal LVEF*	45 (90%)	39 (78%)	0.17
Mean indexed LA volume	33.0 ± 8.7	32.7 ± 9.3	0.86
Beta-blocker	13 (26.0%)	18 (36.0%)	0.38
Class Ic anti-arrhythmic	22 (44.0%)	25 (50.0%)	0.68
Class III anti-arrhythmic	8 (16.0%)	5 (10.0%)	0.99
Oral anticoagulant	31 (62.0%)	29 (58.0%)	0.83

^*^Normal LVEF = EF ≥ 50%. ^**^All cases of valvular heart disease consisted of mitral insufficiency. *LA*, left atrium; *LVEF* left ventricular ejection fraction; *TIA* transient ischemic attack. There were no statistically significant differences among the two arms of the study.

### Procedural details

Procedural characteristics are reported in Table 2. Acute isolation was achieved in all veins in both groups. There were no significant differences in the ablation parameters concerning PVI during CB-A (Table 2). None of the patients presented PNI during the PV ablation.

**Table 2:** Procedural details among study arms

	PVI and SVC isolation (*n* = 50)	PVI alone (*n* = 50)	*p* value
Time to reach SVC isolation (s)	36.7 ± 29.0	NA	NA
Temperature at SVC isolation (°C)	− 35 ± 7	NA	NA
SVC isolation fluoroscopy time (min)	1.6 ± 0.8	NA	NA
Right atrium dwell time (min)	19.5 ± 2.1	NA	NA
Procedure time (min)	88.7 ± 13.6	70.1 ± 15.2	< 0.001
Total fluoroscopy time (min)	25.1 ± 8.4	22.9 ± 12.0	0.29
LSPV number of freezes	1.4 ± 0.4	1.3 ± 0.3	0.16
Minimal temperature (°C)	− 54.0 ± 5.2	− 53.1 ± 4	0.37
LIPV number of freezes	1.3 ± 0.3	1.3 ± 0.2	0.89
Minimal temperature (°C)	− 49.3 ± 5.5	− 48.5 ± 4.4	0.42
RSPV number of freezes	1.2 ± 0.3	1.3 ± 0.4	0.16
Minimal temperature (°C)	− 55.3 ± 5.5	− 54.6 ± 6.0	0.54
RIPV number of freezes	1.2 ± 0.4	1.2 ± 0.3	0.99
Minimal temperature (°C)	− 52.0 ± 6.2	− 50.1 ± 5.8	0.12

*NA* not available. There were no statistically significant differences among the two groups of the study regarding procedural data.

### Superior vena cava isolation

In the PVI and SVCi group, electrical activity was documented in all SVCs prior to ablation. Spontaneous triggers arising from the SVC were observed in four patients. Procedural details of SVCi are reported in Table 2. In 47 (94.0%) patients, a 180-s freeze was accomplished in the SVC, and three patients had at least 120-s of freezing application (6.0%) because of transient or impending PNI. The presence of RTR during SVCi was detected in 42 (84.0%) patients. The mean time to SVCi was 36.7 ± 29.0 s and temperature at SVCi was − 35 ± 7 °C. The mean time to reach − 40 °C was 41.0 ± 11.5 s and temperature at 60 s was − 40 ± 5 °C, while minimum reached temperature was − 42.5 ± 8 °C. Mean SVC diameter was 22.0 ± 3.9 mm. There was no correlation between the ablation parameters and presence or absence of RTR during SVCi. There was no correlation between the ablation parameters and SVC diameter. The diameter of the SVC was bigger in patients with RTR in SVC during ablation (*p* = 0.04, OR = 1.64, 95%CI = 1.29–1.90). During SVCi, two patients presented transient PNI during cryotherapy. Phrenic nerve activity returned to normal before the end of the procedure. Also, one patient experienced impending PN damage with decrease of diaphragmatic contraction. Interruption of the application instantaneously led to complete resumption of phrenic activity. SVCi was accomplished with one application in all patients as there was no case with SVC reconnection during the 15-min waiting period. In our series, all patients exhibited SVC isolation as absence of electrical activity as a result of ablation. We did not observe dissociated activity from the SVC in any patient. No patient presented sinus node injury. No minor or major complication related with the procedure including access site and persistent PN paralysis. In univariate Cox regression of the SVCi and PVI group, none of the SVC ablation parameters, SVC diameter, nor presence of RTR was associated with recurrence of ATs (Table 4).

### Follow-up

**Fig. 3:**
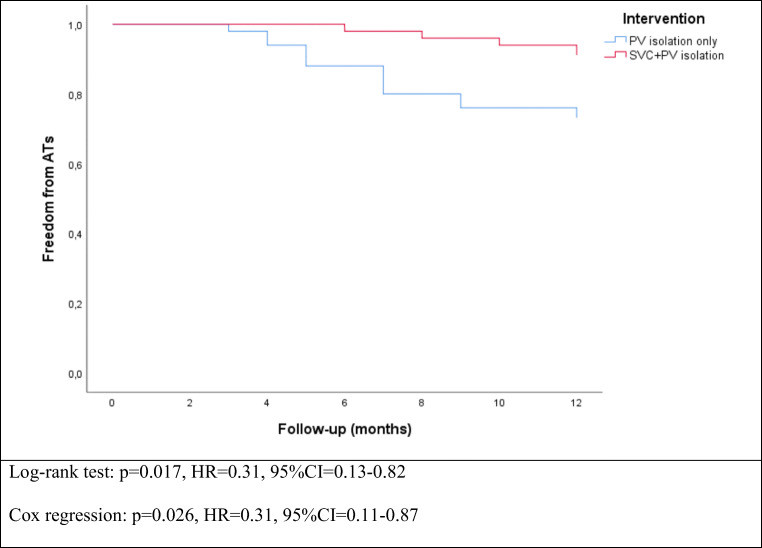
Freedom from ATs recurrence among the study arms.

At 12-month follow-up, considering a blanking period of 3 months, recurrence in the SVC and PV isolation group was 10% (5 patients), while in the standard PVI only group was 28% (14 patients). Contingency table with statistical significance is reported in Table 3. Kaplan-Meier survival curves reporting each group’s arrhythmia-free survival rates are presented in Fig. 3. In the total population, in univariate Cox regression analysis of freedom from ATs recurrence, presence of SVCi was associated with better outcomes, while increased indexed LA volume was associated with worse outcomes (Table 5). However, in multivariate Cox regression analysis of freedom from ATs recurrence in the total population, only presence of SVCi was associated with better outcomes (Table 5).

**Table 3:** Contingency table with incidence of ATs recurrence at 12 months.

	No ATs recurrence	With Ats recurrence
Standard PVI only	36 (72%)	14 (28%)
SVC + PV isolation	45 (90%)	5 (10%)

Fisher’s exact test: *p* = 0.039, OR = 0.80, 95%CI = 0.63–0.96

Binary logistic regression: *p* = 0.027, OR = 0.28, 95%CI = 0.09–0.86

In patients with SVC and PV isolation, freedom from ATs rates at 6 and 12 months was 98% and 90%, respectively (Fig. 3). In this group, there were 5 cases of ATa recurrences, of which one case was in the form of atypical left atrial flutter and in four cases in the form of AF, solved with a roofline, and re-isolation of the right inferior PV, respectively. Remarkably, the SVC was still electrical isolated, and no SVC stenosis was documented in the three patients who experienced a repeat ablation procedure. In univariate Cox survival regression of the PVI and SVC isolation group, none of the SVC ablation parameters, SVC diameter, nor existence of real-time recording, was associated with ATs recurrence (Table 4). However, SVC diameter and time to SVCi presented a borderline significance. In patients with PV isolation only, freedom from ATs rates at 6 and 12 months was 92% and 72%, respectively. Recurrence was in the form of AF in 9 cases, and atypical left atrial flutter in 5 cases (Table 5).

**Table 4:** Univariate Cox regression analysis of freedom from atrial fibrillation recurrence in PVI and SVC isolation group using data regarding SVC isolation (time to AT recurrence is the dependent variable)

SVC and PV isolation (*n* = 50)	*p*	HR	95%CI
SVC diameter (mm)	0.07	1.43	0.97–1.87
SVC freeze duration (s)	0.88	0.99	0.91–1.10
SVC real time recording	0.11	1.31	0.82–1.73
Time to SVC isolation (s)	0.08	1.34	0.92–1.76
Temperature at SVC isolation (s)	0.28	1.28	0.70–1.92
Time to reach − 40 °C (s)	0.40	1.09	0.93–1.25
Temperature at 60 s (°C)	0.45	1.20	0.80–1.61
Minimum reached temperature (°C)	0.52	1.17	0.90–1.45

**Table 5:** Univariate and multivariate Cox regression analysis of freedom from atrial tachycardia recurrence (time to AT recurrence is the dependent variable)

Total population (100 patients)	Univariate analysis			Multivariate analysis		
	*p*	HR	95%CI	*p*	HR	95%CI
Male gender	0.99	1.00	0.60–1.72			
Age (years)	0.55	0.95	0.89–1.10			
BMI	0.32	1.05	0.95–1.12			
CHA2-DS2-VASc score	0.17	1.56	0.88–2.10			
LVEF (%)	0.20	0.96	0.90–1.01			
AF duration (months)	0.12	1.30	0.90–1.70			
Indexed LA size (ml/m^2^)	0.03	1.15	1.05–1.28	0.06	1.12	1.00–1.24
SVC isolation	0.026	0.31	0.11–0.87	0.04	0.78	0.64–0.89

*At*, atrial tachycardia; *BMI*, body mass index; *CI*, confidence interval; *HR*, hazard ratio; *LA*, left atrium; *LVEF*, left ventricle ejection fraction; *SVC*, superior vena cava

## Discussion

To the best of our knowledge, this is the first study that assessed the ATs freedom after 1-year follow-up of CB ablation technique for SVCi after PVI compared to PVI alone. The main finding of our study is that ablation of the SVC in addition to PVI significantly improved outcomes in patients affected by PAF.

Pulmonary vein isolation is the cornerstone of atrial fibrillation ablation, and it eliminates the majority of the triggers for the commencement and maintenance of AF. However, a considerable percentage of patients will present recurrence with ATs following the procedure. Up to 28% of AF patients might exhibit non-PV foci. Among the non-PV foci that might be up to 28% of AF patients, and the SVC is certainly the most common source of ectopy potentially triggering AF [9].

Recently, Santangeli et al [10] described the location and incidence of non PV foci based on their large experience in AF ablation. Interestingly, non-PV foci in the setting of PAF seemed to be mostly originating from the SVC, Eustachian ridge, ligament of Marshall, crista terminalis, coronary sinus, and in proximity of the mitral valve. In a recent article by Hayashi et al. [11], the rate of PAF elicited by non PVI foci was significantly higher than in the abovementioned paper. Specifically, Hayashi concluded that the SVC was the location were non-PV foci could be more frequently recorded. Similar findings were reported by Kawai et al. [12]. The authors observed that non PV foci could be elicited pharmacologically in equal proportions from the SVC and from LA structures. However, these findings were extrapolated from 431 patients having undergone ablation for either PAF or more advanced stages of the disease. Interestingly, the presence of non-PV triggers in the LA was only significantly correlated with persistent or long-standing persistent AF. In accordance with the above-mentioned papers, the SVC played a pivotal role in triggering PAF. The arrhythmogenic properties of the SVC could be justified by the shared embryologic origin of the sinus node (SN) [13]. Therefore, isolation of this structure in addition to PVI could be a critical step to increase the success rates and freedom from arrhythmia recurrence [1, 2, 9, 14–16].

As recently reported, during PVI with the CB can produce parasympathetic modulation [17, 18]. Reports seem to indicate that cardiac denervation caused by ablation might lead to enhanced freedom from AF [19]. In this setting, the ablation of the SVC with the large CB, even not directly, can reach the epicardial ganglionated plexi, between the aortic root just above the right upper pulmonary vein and the superior vena cava [20], and consequently contribute to further parasympathetic modulation. This phenomenon might have played a role in improving clinical outcome if compared to PVI alone.

Ablation of the SVC can be challenging to achieve due to the vicinity of the SN and the PN. In this setting, the primary concern is the inadvertent injury of the right phrenic nerve. Phrenic nerve injury is known to be the most common complications related to the CB ablation technique [4, 21–23]. Radio frequency technique permits to confirm the location of phrenic nerve by local high pacing and avoiding with ablation only that site. The latter can give RF a theoretical advantage to avoid PNI with respect to the CB approach.

Different techniques such as palpation of the diaphragmatic excursion, diaphragmatic compound motor action potentials (CMAPs) monitoring, and the “double stop” strategy have been proposed to avoid PNI [5]. In our study, we monitored the excursion of the diaphragm by manually palpating the abdomen during pacing of the PN while ablating the right sided PVs and the SVC. One benefit of pacing the PN via a subclavian access is to guarantee the total occlusion of the SVC with the CB. This avoids the likely formation of gaps caused by the presence of a pacing catheter positioned through a femoral access and reaching the SVC via the right atrium [24]. In our study, complete recovery of diaphragmatic contraction occurred before the end of the procedure in all patients exhibiting PNI during the ablation of the SVC.

Damage to the SN has been described as a potential complication of SVCi [25]. In our series, inappropriate sinus tachycardia (ISNT) or damage to the SN was not observed. The SVC was isolated with a single freeze in all patients, thus potentially minimizing collateral damage to critical structures. Future randomized studies are required to conclude that, isolation of the SVC in addition to PVI will improve clinical outcome.

Finally, given the good safety profile and the increased success rate, off late, we are progressively starting to perform SVC isolation in addition to PV isolation as standard procedure in all AF patients undergoing PVI with the CB in our center

## Study limitations

This study has several limitations. The study is retrospective in nature and single center. Larger randomized studies are required to confirm our results. Pharmacological testing was not made to systematically examine non-PV triggers. Although no patient exhibited clinical symptoms potentially related to significant SVC stenosis, the incidence of this complication might have been underrated as no post procedural imaging exam was systematically performed. In addition, in our standard practice, we do not routinely perform pacing from the Achieve catheter after isolation to verify exit block as unidirectional block appears to be very rare following ablation [26]. Therefore, the incidence of unidirectional block might have been underestimated. We emphasize that the results are far from generalizable.

## Conclusion

Superior vena cava isolation with the CB in addition to PVI might improve freedom from ATs when compared to PVI alone at a 1-year follow-up.
